# HCMV Protein LUNA Is Required for Viral Reactivation from Latently Infected Primary CD14^+^ Cells

**DOI:** 10.1371/journal.pone.0052827

**Published:** 2012-12-26

**Authors:** Lisa R. Keyes, Danna Hargett, Melisa Soland, Mariana G. Bego, Cyprian C. Rossetto, Graca Almeida-Porada, Stephen St. Jeor

**Affiliations:** 1 Department of Microbiology and Graduate Program in Cell and Molecular Biology, University of Nevada, Reno, Nevada, United States of America; 2 Lewis Thomas Laboratory, Department of Molecular Biology, Princeton University, Princeton, New Jersey, United States of America; Lisbon University, Portugal

## Abstract

Human cytomegalovirus (HCMV) is a member of the *Herpesviridae* family that infects individuals throughout the world. Following an initial lytic stage, HCMV can persist in the individual for life in a non-active (or latent) form. During latency, the virus resides within cells of the myeloid lineage. The mechanisms controlling HCMV latency are not completely understood. A latency associated transcript, UL81-82ast, encoding the protein LUNA (Latency Unique Natural Antigen) was identified from latently infected donors *in vivo*. To address the role of the UL81-82ast protein product LUNA, in the context of the viral genome, we developed a recombinant HCMV bacterial artificial chromosome (BAC) that does not express LUNA. This construct, LUNA knockout FIX virus (FIX-ΔLUNA), was used to evaluate LUNA's role in HCMV latency. The FIX-ΔLUNA virus was able to lytically infect Human Fibroblast (HF) cells, showing that LUNA is not required to establish a lytic infection. Interestingly, we observed significantly higher viral copy numbers in HF cells infected with FIX-ΔLUNA when compared to FIX-WT virus. Furthermore, FIX-WT and FIX-ΔLUNA genomic DNA and transcription of UL81-82ast persisted over time in primary monocytes. In contrast, the levels of UL138 transcript expression in FIX-ΔLUNA infected HF and CD14^+^ cells was 100 and 1000 fold lower (respectively) when compared to the levels observed for FIX-WT infection. Moreover, FIX-ΔLUNA virus failed to reactivate from infected CD14^+^ cells following differentiation. This lack of viral reactivation was accompanied by a lack of lytic gene expression, increase in viral copy numbers, and lack of the production of infectious units following differentiation of the cells. Our study suggests that the LUNA protein is involved in regulating HCMV reactivation, and that in the absence of LUNA, HCMV may not be able to enter a proper latent state and therefore cannot be rescued from the established persistent infection in CD14^+^ cells.

## Introduction

Human cytomegalovirus (HCMV) is a ubiquitous virus affecting all geographic locations and socioeconomic groups. Infection is normally asymptomatic, except in individuals who have an impaired or immature immune response. Following an initial infection, the virus enters a latent state within cells of the myeloid lineage where it is protected from the immune system ([1] and as reviewed in [2]). Naturally infected CD14^+^ cells can harbor latent HCMV DNA [3]–[5], which is capable of reactivating after differentiation into dendritic cells or macrophages [3], [6]–[10]. Despite recent progress in the field, HCMV latency and reactivation still lacks complete molecular characterization.

The hallmarks of a latent infection most prominently include the absence of the expression of viral transactivators, the Immediate Early (IE) genes and consequently structural protein production and lack of infectious viral particles released. Several models have been tested to study HCMV latency *in vitro*. CD34^+^, CD33^+^ granulocyte macrophage progenitors, and CD14^+^ cells are typically used as *in vitro* models for HCMV latency [3]–[5], [8], [11]–[14]. Furthermore, it has been suggested that a specific profile of viral gene expression must be met in order for the virus to become latent, and maintain this latent state [8], [10], [15]–[18]. Expression of UL81-82ast transcript and its protein product LUNA (Latency Unique Natural Antigen) may contribute to latency along with expression of UL138 ORF, UL111.5A, and US28 genes [12], [13], [19]–[21]. However, transcription from all these regions can be found in both a lytic and latent infection.

UL81-82ast was identified from a cDNA library of monocytes isolated from a healthy HCMV seropositive donor, in the absence of Immediate Early (IE) gene expression. The transcript was identified in all HCMV seropositive healthy donors, but not in seronegative ones [22]. LUNA is conserved among all the HCMV isolates, as well as in CMV isolated from chimpanzees, but is not found in any other herpes virus [19]. During lytic infection with a low passage clinical isolate, the transcript is present in human fibroblast (HF) cells early after infection [3], [19]. On the other hand, the transcript is stably expressed throughout latency in vitro [3], [19]. Furthermore, the LUNA protein has been shown to be made *in vivo*, as evidence by the presence of LUNA specific antibodies in the sera of all HCMV seropositive, but not seronegative donors [23]. To date, the LUNA protein has not been further characterized in terms of its significance during lytic replication or latency.

In this study, using a LUNA deficient virus, we have determined that the LUNA protein is an essential component for HCMV reactivation. In the absence of LUNA, HCMV was still able to lytically infect HF cells, and persist in infected primary CD14^+^ cells. However, FIX-ΔLUNA virus failed to reactivate from CD14^+^ cells following differentiation. This study demonstrates that the LUNA protein is a key player during HCMV reactivation.

## Results

### Generation of a LUNA knockout HCMV FIX-BAC

**Figure 1:**
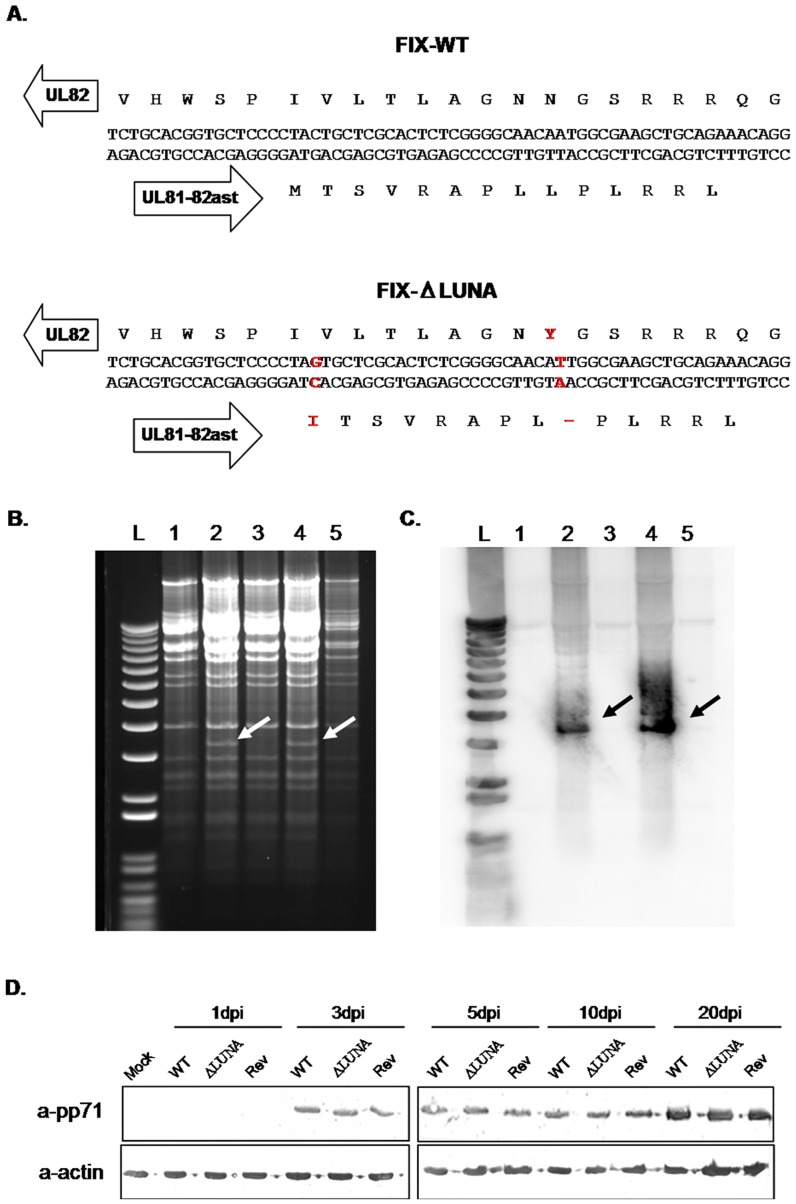
LUNA deletion mutants do not disrupt pp71. A) Sequence of the start codon region for UL8-82ast in FIX-WT and FIX-ΔLUNA viruses. The upper strand represents the coding frame for UL82 and its translation, the bottom strand represents the coding frame for UL81-82ast and its translation. Red-labeled nucleotides and amino acids indicate those that changed after mutagenesis. B) Hind III digest of FIX-BAC-WT (lane 1), FIX-BAC-(GalK-KAN)-A (lane 2), FIX-BAC-ΔLUNA (lane 3), FIX-BAC-(GalK-KAN)-B (lane 4) and FIX-BAC-Rev (lane 5). FIX-BAC-(GalK-KAN)-A contains the Galk-Kan insert before the mutation; FIX-BAC-(GalK-KAN)-B is the re-introduction of the insert in the process of creating the revertant. C) Southern blot of the digest in (B) with a GalK specific probe. The Galk-Kan insert is indicated by the arrow. D) HF cells were infected with FIX-WT, FIX-ΔLUNA or FIX-Rev respectively over a 20day time course. The presence of the pp71 protein was detected via western blotting. 20 µg of protein was added per well, samples were loaded in the following order for each time point: FIX-WT, FIX-ΔLUNA and FIX-Rev. Lane 1: Mock HF, lanes 2–4: HF 1 dpi, lanes 5–7: HF 3 dpi, lanes 8–10: HF 5 dpi, lanes 11–13: HF 10 dpi, lanes 14–16: HF 20 dpi. Blots were incubated with primary antibodies goat anti-pp71 (1∶500) and mouse anti-actin (1∶10,000) and secondary antibodies anti-goat or anti-mouse IgG HRP (1∶1000).

In order to create a LUNA knockout virus, we introduced two point mutations into the FIX-BAC genome using BAC mutagenesis [24]. The first mutation altered the LUNA start codon, and the second introduced a stop codon 20 nt (nucleotides) downstream (Fig. 1A). A GalK-Kan cassette flanking the LUNA start site was inserted in the FIX-BAC-WT and positively selected for with kanamycin. The insertion was analyzed via Hind III digest and Southern blot analysis with a GalK specific probe (Fig. 1B and C). The expected cleavage pattern differences between FIX-BAC-WT and FIX-BAC-(GalK-Kan) were observed. The desired mutations were incorporated and confirmed by restriction digest and the loss of GalK-Kan cassette (Fig. 1B and C). This resulted in the generation of a FIX-BAC genome containing two specific point mutations in the LUNA open reading frame (ORF). The resulting virus was termed FIX-ΔLUNA. Using the above-mentioned procedure, we also developed a revertant virus (FIX-Rev) in which we replaced the two point mutations with their original sequence. The FIX-Rev virus was used to demonstrate that the effects of the FIX-ΔLUNA virus were due specifically to the point mutations, and not the overall mutagenesis process. Only the first mutation of the LUNA ORF altered the pp71 protein coding sequence (UL82), changing the asparagine in position 476 to a tyrosine (Fig. 1A), both are polar amino acids. To date, no known function has been mapped to this site. To verify that the LUNA mutation induced UL82 amino acid change did not inhibit UL82 expression, we tested for the presence of UL82 transcript (using reverse transcription PCR, RT-PCR) and protein expression in HF cells. pp71 protein expression was monitored in HFs only, as cells latently infected with HCMV do not express structural proteins like pp71 [25]–[28]. pp71 was expressed in similar levels in FIX-WT, FIX-ΔLUNA and FIX-Rev infected HF cells at different time points, starting at 3 dpi and maintaining over a 20 d time course (Fig. 1D).

### LUNA is not required to establish or maintain a productive infection in HF cells, or a non-productive infection in CD14^+^ cells

**Figure 2:**
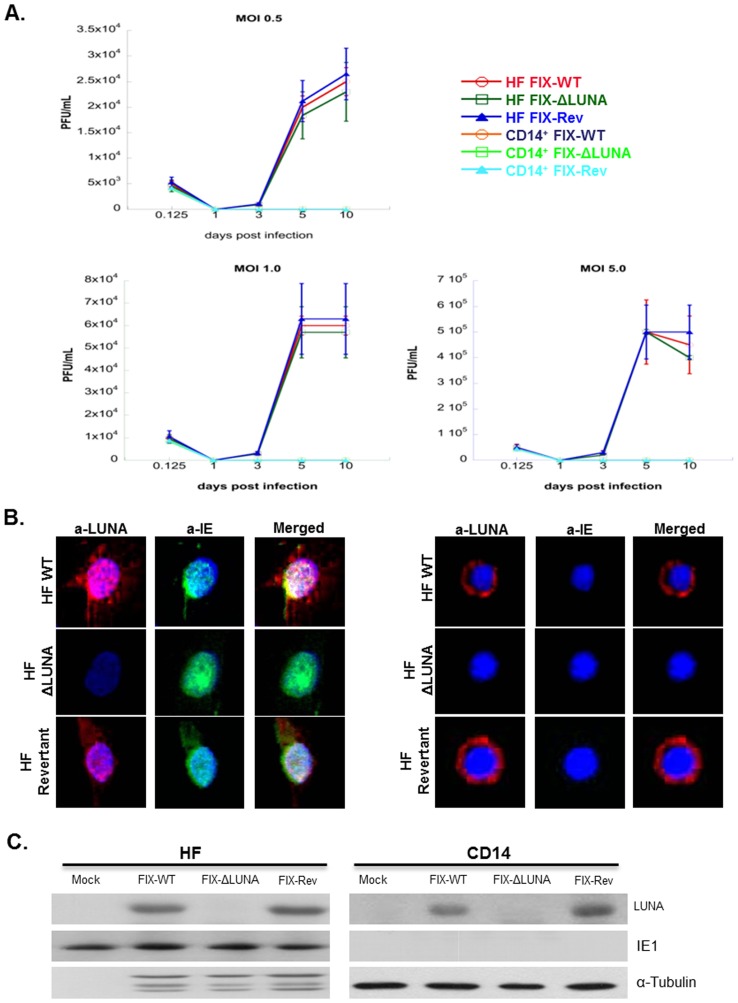
The LUNA protein is dispensable for growth in HF infected cells and to maintain latent infection in CD14^+^ cells. A) HF and CD14^+^ cells were infected at an MOI = 5, MOI = 1, or MOI = 0.5 with either FIX-WT, FIX-ΔLUNA or FIX-Rev. Supernatants from each infection were collected at the indicated time points and titered via standard plaque assay. Samples were tested in triplicate. Student *t* test analysis determined no significant difference among the viral growth of FIX-WT vs. FIX-Rev or FIX-ΔLUNA in either HF cells or in CD14^+^ cells (p>0.5 in both cell lines). B) HF and CD14^+^ cells were infected at an MOI = 3 with either FIX-WT, FIX-ΔLUNA, or FIX-Rev. Samples were fixed at 1 dpi and stained with rabbit anti-LUNA and Alexa Fluor 594 anti rabbit IgG (red) for LUNA detection, and mouse anti-IE primary antibodies along with Alexa Fluor 488 anti-mouse IgG (green). Cell nuclei were stained with DAPI. C) HF and CD14^+^ cells were infected at an MOI = 3 and protein was harvested at 1day post infection for western blot analysis. Proteins were detected using a rabbit anti-LUNA monoclonal antibody (1∶500), mouse anti-IE1 monoclonal antibody (1∶100) and mouse anti-tubulin as a loading control.

Primary CD14^+^ cells were isolated from peripheral blood mononuclear cells (PBMCs) and flow cytometry was performed to test the purity of the isolated population (Fig. S1). Human fibroblast and primary CD14^+^ cells were used as models for lytic and latent HCMV infections, respectively. Viral growth curves from FIX-WT, FIX-ΔLUNA, and FIX-Rev infected HF cells and CD14^+^ cells at different Multiplicities of Infection (MOIs) are shown in Fig. 2A. Production of infectious virions increased over time in HF cells, consistent with a lytic infection. Furthermore, using a Student *t* test, no statistically significant difference among the viral growth curves was observed when comparing FIX-WT or FIX-Rev to FIX-ΔLUNA virus in HF cells (FIX-WT:FIX-ΔLUNA p>0.5, FIX-Rev:FIX-ΔLUNA p>0.8) (Fig. 2A). On the other hand, infection of CD14^+^ cells with FIX-WT, FIX-ΔLUNA, or FIX-Rev viruses showed no infectious viral particles being released. Immunofluorescence was used to verify that the mutations made to the UL81-82ast locus resulted in abolishment of LUNA protein expression. FIX-WT, FIX-Rev, and FIX-ΔLUNA infected HF cells or CD14^+^ cells were fixed at 1 dpi and stained for IE and LUNA proteins to monitor infection and LUNA protein expression. FIX-WT and FIX-Rev infection of both HF cells and CD14^+^ cells resulted in expression of the LUNA protein, but not in FIX-ΔLUNA infection (Fig. 2B). IE protein expression was detected in HF cells as expected during lytic replication, however, consistent with previous reports, no IE protein was detected in latently infected CD14^+^ cells [11] (Fig. 2B). In order to further verify these results, protein was harvested from HF and CD14^+^ cells infected at an MOI = 3 at 1day post infection. Western Blot analysis was used to assay the protein samples for the presence/absence of the LUNA protein and IE protein in each FIX-WT, FIX-Rev, and FIX-ΔLUNA infected HF cells or CD14^+^ cells. Similar levels of LUNA protein were observed in both FIX-WT and FIX-Rev infected HF and CD14^+^ cells, whereas LUNA protein was not detected in either cell type when infected with FIX-ΔLUNA (Fig. 2C). Consistent with our IFA data (Fig. 2B) the levels of IE protein remained constant between FIX-WT, FIX-Rev and FIX-ΔLUNA infected HF cells, and were absent in infected CD14^+^ cells (Fig. 2C). Overall, our results indicate that FIX-ΔLUNA and FIX-Rev grow to similar titers as FIX-WT, indicating that the mutagenesis did not have any negative effects on viral growth/production, and that LUNA is dispensable for lytic replication.

### FIX-ΔLUNA fails to express lytic transcripts following differentiation of CD14^+^ cells with IL6

**Figure 3:**
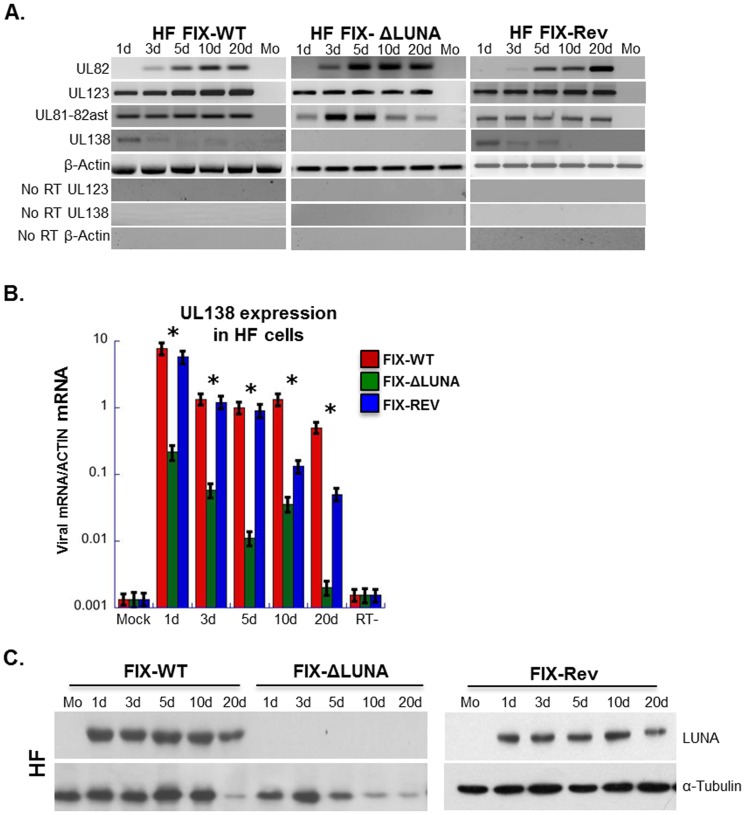
LUNA protein expression is not required for lytic gene expression, but does augment the expression of the UL138 transcript. HF cells infected at an MOI = 1 with either FIX-WT, FIX-ΔLUNA and FIX-Rev were harvested at the indicated time points for either RNA or protein analysis. A) Expression of viral RNAs. Total RNA was collected over a 20 d time course together with mock RNA. From all the collected RNA samples cDNA was synthesized and amplified using UL82, UL123, UL81-82ast, UL138 and β-actin specific primers. Negative images were used to visualize weaker bands. B) qRT-PCR analysis was used to assay gene expression of UL138 at the indicated times post infection. Viral mRNA was normalized to actin. Asterisks indicate significant changes (p value<0.05), as determined by student t test. C) Protein was isolated from infected HF cells at the indicated time points and used for western blot analysis. Blots were probed with rabbit anti-LUNA (1∶500) or α-tubulin as a loading control. All samples were tested in triplicate. Abbreviations used: d; days post infection, Mo; mock.

Further characterization of gene expression from FIX-WT, FIX-ΔLUNA and FIX-Rev was conducted in HF cells and primary CD14^+^ cells. In order to determine the type of infection (lytic or latent) observed per cell type, we analyzed the presence of lytic and latent viral transcripts by RT-PCR using primers described in Table 1. Three biological replicates for each time point/cell type were isolated and assayed. During a lytic infection, viral genes are temporally expressed, beginning with expression of the Immediate Early (IE) genes (UL123) which drive expression of the Early genes, followed by expression of the Late genes (such as UL82) [29], [30]. On the other hand, a typical latent infection is characterized by the lack of structural gene expression, and expression of a subset of latency associated genes including UL81-82ast and UL138 [1], [3], [19], [31], [32]. Consistent with lytic infection, UL123 transcript expression was detected throughout infection in FIX-WT, FIX-ΔLUNA and FIX-Rev infected HF cells (Fig. 3A). The UL82 transcript (Fig. 3A and B) was detected in FIX-WT, FIX-ΔLUNA and FIX-Rev infected HF cells starting at 3 dpi (Fig. 3A). The presence of this transcript and pattern of expression in FIX-ΔLUNA infected HF cells mimics that of both a FIX-WT and FIX-Rev infection, suggesting that our mutations to the antisense strand (UL81-82ast) did not impair UL82 expression. Analysis of gene expression of infected HF cells revealed that UL81-82ast transcripts were consistently detected when infecting with FIX-WT, FIX-ΔLUNA or FIX-Rev through 20 dpi (Fig. 3A). UL81-82ast transcript is present throughout infection consistent with the previously observed long term expression of the LUNA protein [19]. Furthermore, while UL138 is detectable in FIX-WT and FIX-Rev infected samples, we were unable to detect the UL138 transcript at any time point post infection in the FIX-ΔLUNA infected HF cells using RT-PCR (Fig. 3A). This result led us investigate the lack of UL138 expression further using quantitative real-time PCR (qRT-PCR). Using qRT-PCR we found that the UL138 transcript is expressed in HF cells infected with FIX-ΔLUNA, however, on average UL138 expression was 100 fold lower when compared to FIX-WT or FIX-Rev infected HF cells (Fig. 3B). This suggests that the LUNA protein may in fact enhance the expression of UL138. Although the LUNA transcript is present, the mutations made eliminate LUNA protein expression. To confirm that the LUNA protein was not being expressed at any time during infection we used western blot analysis to detect the presence or absence of the LUNA protein over the same 20 day time course. The LUNA protein was detected in both infected FIX-WT and FIX-Rev HF cells at similar levels overtime, however, LUNA protein was absent in FIX-ΔLUNA infected HF cells (Fig. 3C). Taken together, this data suggests that the LUNA protein is not required for lytic gene expression in HF cells, but does augment the expression of the UL138 transcript during lytic infection.

**Table 1:** Primers for BAC mutagenesis and RT-PCR.

Primer Name	F/R^a^	Sequence (5′-3′)
LUNA-Mut-F	F	5′-GTCCCGGGCACTGATCCTGACCGGAC
		AAAGACGTCGAAGCGGTTACAACGGGGC
		TCTCACGCTCGTGATCCCCTCGTGGCAC
		GTCTTTGCGAGCCTTGACGACTTGGTAC-3′
LUNA-Mut-R	R	5′-GTACCAAGTCGTCAAGGCTCGCAAAGAC
		GTGCCACGAGGGGATCACGAGCGTGAGAG
		CCCCGTTGTAACCGCTTCGACGTCTTTGTC
		CGGTCAGGATCAGTGCCCGGGAC-3′
Rev-F	F	5′-GTCCCGGGCACTGATCCTGACCGGACAA
		AGACGTCGAAGCGGTAACAACGGGGCTC
		TCACGCTCGTCATCCCCTCGTGGCACGTC
		TTTGCGAGCCTTGACGACTTGGTAC-3′
Rev-R	R	5′-GTACCAAGTCGTCAAGGCTCGCAAAG
		ACGTGCCACGAGGGGATGACGAGCGT
		GAGAGCCCCGTTGTTACCGCTTCGACGTCTTT
		GTCCGGTCAGGATCAGTGCCCGGGAC -3′
GalK-F	F	5′-CTTAACGGTCAGGAAGCAG-3′
GalK-R	R	5′-CAGCACTGTCCTGCTCCTTG-3′
β-actin-F	F	5′-AGCAAGAGAGGCATCCTC-3′
β-actin-R	R	5′-TGCGGATGTCCACGTCAC-3′
IE 2A	F	5′-ATGGAGTCCTCTGCCAAGAGAAAGATGGAC-3′
IE 4B	R	5′-CAATACACTTCATCTCCTCGAAAGG-3′
IE 3A	F	5′-GTGACCAAGGCCACGACGTT-3′
IE 3B	R	5′-TCTGCCAGGACATCTTTCTC-3′
UL82	F	5′-GTGGATCCATGTCTCAGGCATCGTCC-3′
UL82	R	5′-GATATCCTAGATGCGGGTCGACT-3′
UL138-F	F	5′-GGTTCATCGTCTTCGTCGTC-3
UL138-R	R	5′-CACGGGTTTCAACAGATCG-3′
LUNA-F	F	5′-TGCATCACGACTCACCGCAC-3′
LUNA-R	R	5′-GGAAGTGGAAGCGGTGCTGA-3′

a F: Forward Primer; R: Reverse Primer.

**Figure 4:**
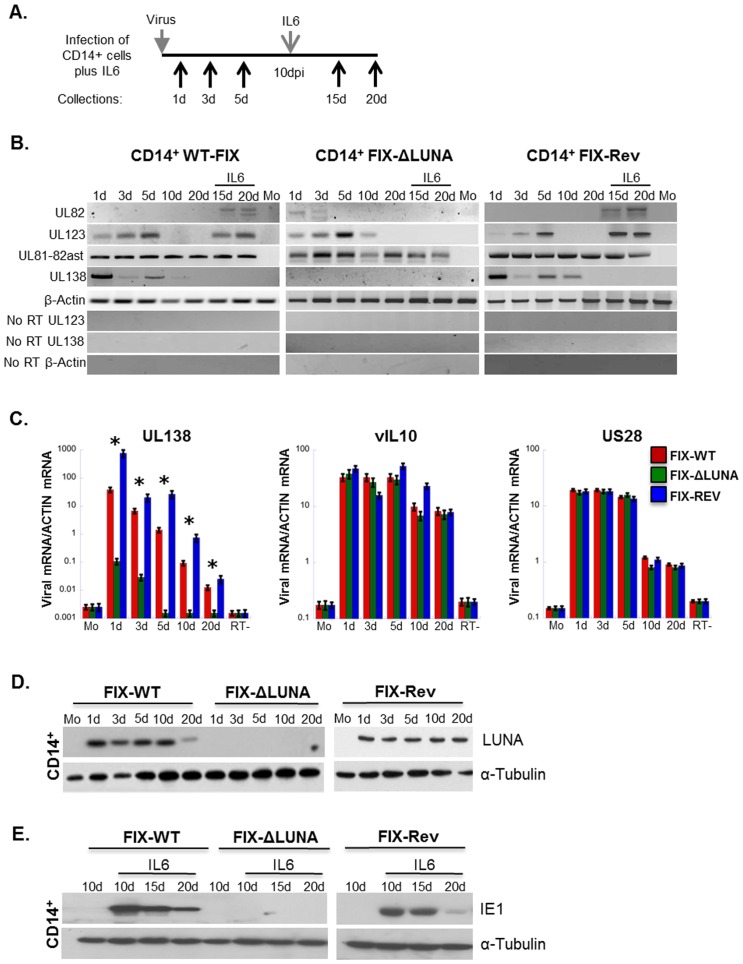
FIX-ΔLUNA infected CD14+ cells fail to express lytic transcripts following IL6 induced differentiation. A) Diagram of the timeline of infection. Infected cells were collected at 1–20 dpi, IL6 was added at 10 dpi to induce cellular differentiation, after which two additional time points were collected. B) Expression of viral RNAs in FIX-WT, FIX-ΔLUNA and FIX-Rev infected CD14^+^ cells (MOI = 1). Total RNA was collected over a 20 d time course together with mock RNA. At 10 dpi CD14^+^ cells were differentiated with IL-6. Five and ten days post differentiation, RNA samples were collected (these samples were termed as 15 d-IL6 and 20 d-IL6). From all the collected RNA samples cDNA was synthesized and amplified using UL82, UL123, UL81-82ast, UL138 and β-actin specific primers. Negative images were used to visualize weaker bands. C) CD14^+^ cells were infected at an MOI = 1 with FIX-WT, FIX-ΔLUNA or FIX-Rev. D–E) Protein was isolated from infected CD14^+^ cells at the indicated time points and subjected to western blot analysis. Blots were probed with either a rabbit monoclonal a-LUNA or a mouse monoclonal a-IE1 antibody along with a-Tubulin as a loading control. qRT-PCR analysis was used to assay gene expression of UL138, vIL10 and US28 at the indicated times post infection. Viral mRNA was normalized to actin. All samples were tested in triplicate. Abbreviations used: d, days post infection., Mo; mock. IL6 was added at 10 dpi.

FIX-WT, FIX-ΔLUNA and FIX-Rev infected primary CD14^+^ cells were analyzed for viral RNA prior to and post IL6-induced differentiation of CD14^+^ cells following protocols previously established [3]. RNA samples were collected at 1 d, 3 d, 5 d, 10 d, and 20 dpi. Alternatively, IL6 was added at 10 dpi to differentiate CD14^+^ cells and trigger HCMV reactivation, and samples were collected at 15 d and 20 dpi (samples were termed as 15 d-IL6 and 20 d-IL6, respectively, Fig. 4A). UL81-82ast transcripts were detected in FIX-WT, FIX-ΔLUNA and FIX-Rev infected CD14^+^ cells throughout the course of infection, including post IL6 induced differentiation (Fig. 4B). In agreement with previous reports [3], [12], [17], UL123 transcripts were detected in FIX-WT and FIX-Rev infected CD14^+^ cells only for the first 5 dpi, while no detectable amplification was observed at the 10 d or 20 dpi time points. Following IL6 induced reactivation of the FIX-WT and FIX-Rev virus, expression of the UL123 transcripts was recovered. On the other hand, in FIX-ΔLUNA infected CD14^+^ cells, UL123 transcripts could be detected up to 10 dpi and no amplification of UL123 was observed following differentiation. As neither UL123 or UL82 transcripts were detected at 10 dpi, we concluded that latency was established in the FIX-WT, and FIX-Rev infected CD14^+^ cells. Both UL123 and UL82 lytic transcripts were detected at later time points during FIX-ΔLUNA infection compared to FIX-WT and FIX-Rev infection. Interestingly, while we observed a single band for UL82 in our HF infected cells, we observed a double band in our infected CD14^+^ cells. It is possible that the presence of these double bands is due to differential expression of splice variants. Typically CD14^+^ cells are not able to support lytic infection, and thus may not be capable of proper expression of this late gene. Furthermore, as the cells differentiate, we may see the product of UL82 as it is first being made in both partially differentiated and fully differentiated cells, which may explain the difference in patterns. These data suggest that either there was a delay in the establishment of latency in FIX-ΔLUNA infected CD14^+^ cells, or that a proper latent infection was not achieved in these cells. To better characterize the delay in latency establishment during FIX-ΔLUNA infection, we analyzed the expression of the UL138 latency associated transcript. Consistent with CD34^+^ hematopoietic stem cell (HSC) infection [12], FIX-WT and FIX-Rev infected CD14^+^ cells expressed UL138 transcripts through 10 dpi. Surprisingly, and in agreement with our data for HF infected cells, FIX-ΔLUNA infection did not result in detectable expression of UL138 in CD14^+^ cells (Fig. 4C) using RT-PCR. The lack of UL138 expression, and apparent lack of reactivation prompted us to more closely examine latent gene expression in CD14^+^ cells infected with FIX-WT, FIX-ΔLUNA or FIX-Rev using qRT-PCR. vIL10 and US28 are also considered latency associated genes [3], and therefore, we sought to determine if the lack of LUNA protein expression had an effect over these two transcripts as it did for UL138. We did not observe any difference in the expression of vIL10 or US28 in CD14^+^ cells infected with either FIX-WT, FIX-ΔLUNA or FIX-Rev (Fig. 4C). However, qRT-PCR analysis of UL138 demonstrated that although UL138 was expressed at low levels throughout infection in CD14^+^ cells infected with FIX-ΔLUNA, expression was between 100–1000 fold less when compared to FIX-WT or FIX-Rev infection, suggesting that the aberrant latency establishment may be due to insufficient levels of UL138 expression. No products were observed in mock-infected cells, indicating specificity of the signal detected and no DNA contamination was observed as amplification of RNA samples with primers against UL123, UL138 or β-actin resulted negative (Fig. 4B). In order to confirm the lack of LUNA protein expression overtime in the presence of the LUNA transcript, we used western blot analysis to detect the presence or absence of LUNA protein. We observed similar levels of the LUNA protein overtime in both FIX-WT and FIX-Rev infected CD14^+^ cells, whereas no LUNA protein was detected in CD14^+^ cells infected with FIX-ΔLUNA (Fig. 4D). As we did not see expression of the UL123 transcripts in CD14^+^ cells infected with FIX-ΔLUNA following IL6 induced differentiation, we performed western blot analysis to confirm the lack of this lytic protein. Following IL6 induced differentiation of CD14^+^ cells and thus reactivation of FIX-WT or FIX-Rev, IE protein expression was observed at 10 d, 15 d, and 20 d post IL6 induction (Fig. 4E). However, no IE protein expression was detected following IL6 induced differentiation of CD14^+^ cells infected with FIX-ΔLUNA at any time point (Fig. 4E). Overall, these results indicate that there is a defect in the ability of FIX-ΔLUNA virus to efficiently establish a latent infection, and furthermore, that virus lacking LUNA protein expression is unable to express lytic transcripts (UL82 and UL123) as well as the IE protein following IL6 induced differentiation, thus preventing viral reactivation.

### FIX-ΔLUNA cannot reactivate after primary CD14^+^ cell differentiation

**Figure 5:**
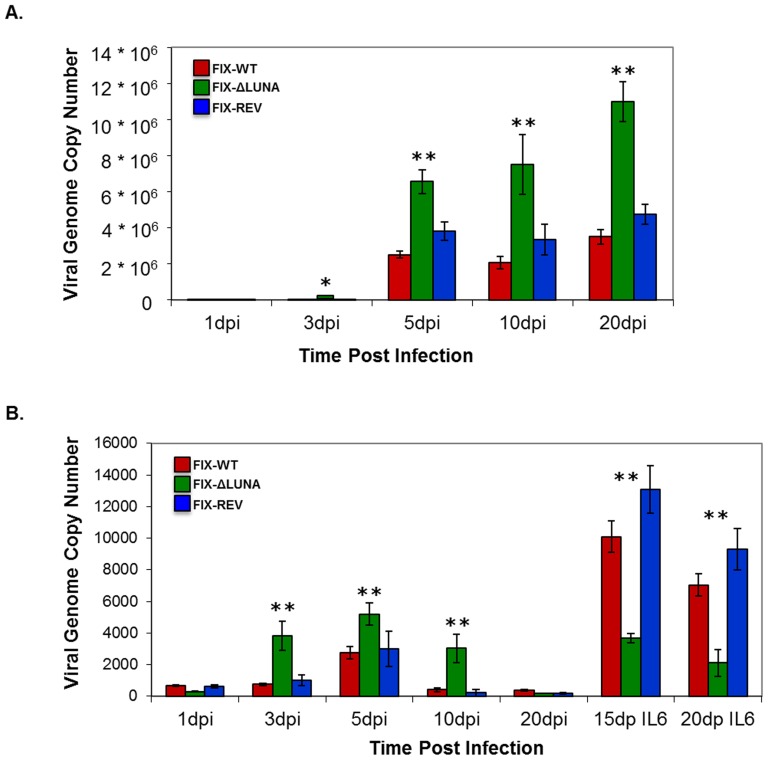
FIX-ΔLUNA virus does not increase viral genome copy number after IL6 induced differentiation of CD14^+^ cells. Viral DNA was quantified by qPCR. A) Total DNA was extracted from FIX-WT, FIX-ΔLUNA, and FIX-Rev infected HF cells over a 20 d time course. B) Total DNA was extracted from FIX-WT, FIX-ΔLUNA, and FIX-Rev infected CD14^+^ cells at 1 dpi, 3 dpi, 5 dpi, 10 dpi, 20 dpi. Differentiation was induced in CD14^+^ cells at 10 dpi by the addition of IL6 and at 5 days (sample termed as 15dpIL6) or 10 days (samples termed as 20dpIL6) total DNA was collected. All samples were run in triplicate. Statistical analysis was done using a Student *t* test comparing FIX-WT vs. FIXΔLUNA or FIX-Rev vs. FIX-ΔLUNA. The * indicates a *P*-value<0.01, and the ** indicates a *P*-value<0.001.

As our analysis of viral transcripts suggested a role for LUNA in reactivation from latency, we tested the ability of the FIX-ΔLUNA virus to replicate viral genomes after stimulation of CD14^+^ cells to differentiate with IL6. Using qPCR, viral copy numbers were monitored over a 20-day time course using primers described in Table 2. As seen in Fig. 5A, viral genome copy numbers increased in FIX-WT, FIX-ΔLUNA, and FIX-Rev infected HF cells overtime, as expected from a lytic infection. Surprisingly, the viral copy numbers were significantly higher in FIX-ΔLUNA infected HF cells than in FIX-WT (p<0.016) or FIX-Rev (p<0.03) infected HF cells at later time points, (∼3 fold at 5 dpi–20 dpi). Moreover, there was no statistically significant difference in viral genome copy number between FIX-WT and FIX-Rev (p>0.5).

**Table 2:** Primers qPCR, and qRT-PCR.

Primer Name	F/R^a^	Sequence (5′-3′)
syGapDH-F	F	5′-ATGGAAATCCCATCACCATCTT-3′
syGapDH-R	R	5′-CATCCTAGTTGCCTCCCCAAA-3′
*β-actin	F	5′-CATTGCCGACGGATGCA-3′
*β-actin	R	5′-GCCGATCCACACGGAGTACT-3′
*UL123	F	5′-GCCTTCCCTAAGACCACCAAT-3′
*UL123	R	5′-ATTTTCTGGGCATAAGCCATAATC-3′
UL138	F	5′-TGCGCATGTTTCTGAGCTAC-3′
UL138	R	5′-ACGGGTTTCAACAGATCGAC-3′
US28	F	5′-TTTGGTGGATCTTTGCCGTG-3′
US28	R	5′-ACGAAAGCACCAAGCATGAGTTC-3′
vIL-10	F	5′-TGTTGAGGCGGTATCTGGAGA-3′
vIL-10	R	5′-CCGTCTTGAGTCCGGGATAG-3′

* primers also used in Figure S2.

As expected, latent infection of primary CD14^+^ cells yielded only marginal levels of HCMV DNA (Fig. 5B). Notably, the viral copy numbers pre-differentiation were also significantly higher in FIX-ΔLUNA infected CD14^+^ cells than in FIX-WT (p<0.04) or FIX-Rev (p<0.05) infected CD14^+^ cells from 3 d to 10 dpi (∼5 fold at 3 d, ∼2 fold at 5 d, and ∼7.5 fold at 10 dpi). High viral copy numbers were maintained up to 10 dpi in FIX-ΔLUNA infected CD14^+^ cells, whereas the viral copy numbers in FIX-WT and FIX-Rev infected CD14^+^ cells remained consistently low. Following differentiation, viral DNA replication was detected as observed by the increased levels of viral DNA in FIX-WT and FIX-Rev infected CD14^+^ cells compared to pre-differentiation levels (Fig. 5B). Nevertheless, differentiation of CD14^+^ cells infected with FIX-ΔLUNA did not generate a significant increase in viral copy number when compared to pre-differentiation viral copy levels (p>0.3). Moreover, there was a ∼3–4 fold decrease in the level of viral copy numbers in FIX-ΔLUNA CD14^+^ cells after differentiation when compared to infection with FIX-WT (p<0.01) or FIX-Rev (p<0.01). In order to further address the lack of reactivation, we used cell dilution PCR to determine the number of available genomes prior to the addition of IL6 stimulus, and thus reactivation. No significant differences in genome numbers were noted between FIX-WT, FIX-Rev and FIX-ΔLUNA infected CD14^+^ cells prior to reactivation (Fig. S2).

**Figure 6:**
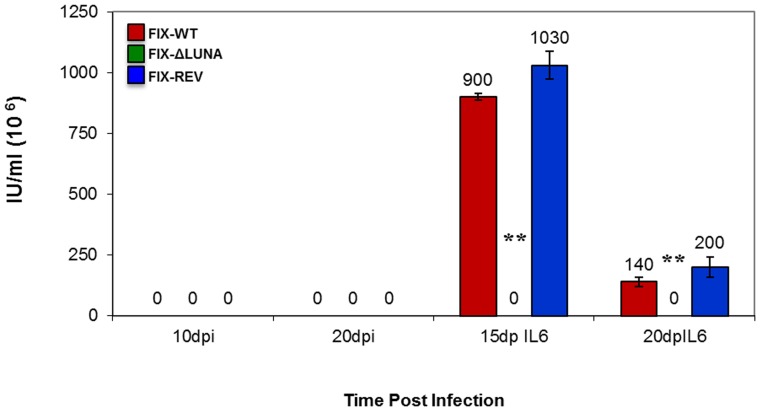
FIX-ΔLUNA viral infection of CD14^+^ cells fail to produce infectious virus after IL6 differentiation. A) Cell lysates plus supernatant from FIX-WT, FIX-ΔLUNA, or FIX-Rev infected cells were collected at 10 dpi and 20 dpi. IL6 was added at 10 dpi; at 5 (15dpIL6) and 10 (20dpIL6) days after differentiation, cell lysates and sups were also collected. The collected lysate/supernatant mix was serially diluted and used to infect HF cells. Two days post infection, cells were fixed with 4× paraformaldehyde, then stained with mouse anti-IE1/2 (1∶250) overnight. Cells were then incubated with Alexa Fluor 488 anti-mouse IgG for 1 hr, and incubated with DAPI for 30 min. IE positive cells were counted to determine the number of infectious units (IU). Only the time points post reactivation showed any significance when comparing FIX-WT or FIX-Rev to FIX-ΔLUNA (10dpIL6: p<3×10^−5^; 20dpIL6: p<0.0006). The ** indicates a *P*-value<0.001.

In order to confirm that reactivation of virus from latently infected CD14^+^ cells resulted in virion production, we conducted an infectious units (IU) assay (Fig. 6). Since the FIX virus is largely cell associated, cell lysates plus supernatant from infected cells were used. This mixture of supernatant plus cell lysate from infected CD14^+^ cells was serially diluted and used to infect HF cells. After 48 hpi, cells were fixed and stained for IE proteins. Each IE positive cell was counted as one infectious unit. No infectious virus was detected from FIX-WT, FIX-Rev and FIX-ΔLUNA infected CD14^+^ cells at 10 dpi and 20 dpi. However, following cellular differentiation after the addition of IL6 to culture media, there was a drastic increase in infectious units production when CD14^+^ cells were infected with FIX-WT or FIX-Rev. In agreement with our previous observations, CD14^+^ cells infected with FIX-ΔLUNA generated no infectious particles following IL6 induced differentiation as a difference when infecting cells with FIX-WT (FIX-ΔLUNA vs. FIX-WT; p<0.0003) or FIX-Rev (FIX-ΔLUNA vs. FIX-Rev; p<0.0003). Taken together, our results indicate that the LUNA protein plays a role in HCMV reactivation in primary CD14^+^ cells.

## Discussion

The LUNA transcript was first isolated from blood samples of latently infected donors [19]. Translation from this transcript was later confirmed *in vivo* [22]. The LUNA promoter was shown to be active during latent and lytic infections, consistent with the reported expression patterns of LUNA *in vitro* [19], [27]. However, the functional relevance of LUNA has not been studied. The data presented here suggests that the LUNA protein is an important factor in the reactivation of HCMV infection in CD14^+^ cells. The FIX-ΔLUNA virus was able to infect both HF cells and CD14^+^ cells, suggesting that the HCMV's ability to infect cells is not dependent on, or regulated by the LUNA protein.

The role of LUNA during productive infection of HF cells still remains obscure. We did not observe any significant difference in the levels or kinetics of immediate early (UL123) and late (UL82) gene expression between the FIX-ΔLUNA infected HF cells and HF cells infected with FIX-WT virus (Fig. 3A). Interestingly we did observe a variation in the levels of UL81-82ast RNA expression over time in HF cells infected with FIX-ΔLUNA, whereas the UL81-82ast RNA expression was consistent over time in HF cells infected with either FIX-WT or FIX-Rev. It is possible that the LUNA protein acts to auto-regulate its own expression, however, further investigation is needed to clarify this phenomenon. Furthermore, FIX-WT, FIX-Rev and FIX-ΔLUNA infection of HF cells produced similar amounts of infectious particles, indicating that LUNA did not have any negative effects on viral growth/production, and that LUNA is dispensable for lytic replication (Fig. 2A). Surprisingly, viral copy numbers were significantly higher in FIX-ΔLUNA infected HF cells (Fig. 5A). UL138 transcript expression was 100 fold lower in FIX-ΔLUNA infected HF cells when compared to FIX-WT and FIX-Rev during lytic infection (Fig. 3A), this data suggests that the LUNA protein may augment the expression of UL138. The UL138 protein localizes to the Golgi, during lytic replication, and is thought to play a potential role in secondary viral envelopment [12]. Further investigation is needed to fully characterize the role of LUNA in regulating UL138 expression and whether this regulation impacts viral replication.

Primary monocytes were used as a latency infection model [3]. Consistent with CD34^+^ infection [17], UL123 (IE1) transcript was transiently expressed during latency (Fig. 3C) in infected CD14^+^ cells. Also in agreement with previous reports, latency associated transcripts (LATs), UL81-82ast, UL138, vIL10 and US28 are also detected after FIX-WT infection (Fig. 3C) [3], [12], [27]. Prototypical latent gene expression patterns, together with the retention of the viral genome (Fig. 5B) and absence of infectious virion production (Fig. 1A and 6) suggest the establishment of a latent infection. Furthermore, FIX-WT latent virus reactivated after CD14^+^ cells were differentiated by IL6 (Fig. 5B and 6). We further characterized the lack of reactivation by monitoring the number of available viral genomes prior to reactivation in FIX-WT, FIX-Rev and FIX-ΔLUNA CD14^+^ cells (Fig. S2), no significant differences were observed indicating that the lack of reactivation is not a result of the lack of available genomes.

LUNA is also not involved in the maintenance of latency as no spontaneous reactivation was seen after infection with FIX-ΔLUNA (Fig. 6). On the other hand, although expression patterns of UL123 and UL81-82ast transcripts were similar to FIX-WT, in FIX-ΔLUNA infected CD14^+^ cells, UL138 transcript expression was between 100 and 1000 fold lower (Fig. 4C). The drastic decrease in UL138 transcript expression in CD14^+^ cells, which is required for latency establishment in CD34^+^ HSC [12], [13], may contribute to the delay in the ability of FIX-ΔLUNA to fully establish a latent infection. Although we observed a 14.6 fold decrease in viral genome copy number in FIX-ΔLUNA infected CD14^+^ cells from 10 dpi to 20 dpi, we did not see a drop in the transcript levels of US28 and vIL-10. As both US28 and vIL-10 are cellular homologs, it is possible that cellular promoter or enhancer elements were maintained in the process of copying the cellular gene into the viral genome. Therefore, transcription of the viral homologue may still be dictated by cellular transcription factors. Levels of cellular transcription factors would not fluctuate with viral genome levels and thus steady expression could be maintained for these specific genes. Furthermore, the mRNA stability of either of these genes under these experimental conditions is unknown. The consistent levels of mRNA seen across the total 20 dpi of time points in infected cells does bolster the argument that US28 and vIL-10 are highly stable under these experimental conditions. The literature suggests that the cellular homolog of US28 is relatively stable [33], therefore, US28 mRNA may also be stable. Although cellular IL-10 is considered to have a short half-life, that of vIL-10 is still unknown. Furthermore, studies have shown that vIL-10 expresses a different splice variant during a latent infection [3], [34]. It may be possible that this splice variant is more stable than its cellular homolog.

Although we did not detect lytic viral transcripts (Fig. 4C), an increase in viral copy number (Fig. 5B), or the production of infectious virus (Fig. 6) by 20 dpi in FIX-ΔLUNA infected CD14^+^ cells, the low levels of UL138 may indicate that FIX-ΔLUNA did not fully establish a latent infection, but possibly a non-productive persistent infection. Moreover, we were unable to reactivate and rescue virus from this non-productive state (Fig. 5B and 6). Even when addition of IL6 was still sufficient to induce differentiation of cells infected with FIX-ΔLUNA, no significant increase in viral genome copy numbers, expression of lytic genes, or production of infectious particles was detected as compared to prior to differentiation. FIX-ΔLUNA infected CD14^+^ cells maintained low levels of viral genome copy numbers similar to those detected prior to IL6 induced differentiation (Fig. 4B), with no indication of viral reactivation. As UL123 gene expression is required for HCMV DNA replication [35], it is highly likely that the failure of FIX-ΔLUNA infected cells to reactivate viral genome replication post IL6 stimulus is a consequence of the failure to re-express the UL123 genes and consequently IE protein (Fig. 4C and 4E). Thus, we can speculate that LUNA may be required to regulate viral gene expression in a non-productive state, as seen by a drastic decrease in UL138 transcript expression during latency and the absence of UL123 gene expression/IE protein expression to drive reactivation in absence of LUNA production. The evidence presented suggests two potential roles for LUNA. LUNA may be important for the proper establishment of latency, through its augmentation of UL138 expression. Also, LUNA may regulate the transition from a non-productive to lytic replication, through its regulation of UL123 gene expression. Indeed, although HCMV factors have previously been categorized as either latent- or lytic-associated, it is perhaps prudent to reconsider these distinctions and acknowledge specific roles in latency establishment, maintenance, and as transition state facilitators.

Our data suggests that LUNA is essential to control both latency-promoting factors, like UL138, and in the ability of HCMV to reactivate lytic replication [12], [13]. We can speculate as to several possible mechanisms for LUNA controlling latency and reactivation. First, LUNA may directly regulate viral gene expression in the latent state. As LUNA may positively regulate UL138 during initial infection, under the conditions of pro-inflammatory signals, LUNA function may also drive the expression of immediate early genes. If latency is not properly established, due to the low levels of UL138, then the virus would not be able to reactivate. Alternatively, LUNA may have a direct effect over UL123 gene expression as well. Second, LUNA may play a role in the development of a latency-specific viral genome structure. Recent studies of infected CD34^+^ cells have provided evidence that latent viral genomes associate with heterochromatin structures [26], [36]. LUNA proteins may play a role in the formation of these structures, which may be altered after differentiation of CD14^+^/CD34^+^ cells. As LUNA is constitutively expressed, it may play a more direct role in unwinding these heterochromatin latency-specific structures in response to differentiation signaling.

HCMV reactivation during immunosuppression can contribute to morbidity and mortality, as well as graft/transplant rejection. Therefore, it is essential to characterize both the cellular and viral aspects of HCMV reactivation in order to design targeted intervention strategies. As the LUNA protein has been shown to elicit an immune response *in vivo*, [23], LUNA may perform a crucial function during HCMV infection. The studies presented here suggest that LUNA functions to aid the proper establishment of a latent infection, as well as the ability to induce viral reactivation. Consequently, therapies against LUNA expression should be developed in order to prevent reactivation of HCMV in transplant recipients and immunocompromised individuals.

## Materials and Methods

### Cells and viruses

Human fibroblast (HF) cells kindly provided by Dr. Greg Pari were grown and maintained in Dulbecco-Modified Eagle Medium (DMEM) supplemented with 10% FBS. Human blood was provided by United Blood Services (Sparks, NV center). Peripheral blood mononuclear cells (PBMCs) were isolated via Ficoll-paque gradients, following manufacturer's recommendations. CD14^+^ cells were isolated from PBMCs using a MACS sorting kit and anti-CD14^+^ microbeads following manufacturer's protocol (Miltenyi Biotech). Purity of CD14^+^ cells was analyzed by flow cytometry as described below. CD14^+^ cells were cultured in DMEM, 20% heat-inactivated FBS, 25 mM HEPES, 50 ng/ml M-CSF, 50 ng/ml SCF, 50 ng/ml G-CSF, 50 ng/ml GM-CSF, and 50 ng/ml IL3; in low adherence plates. Differentiation into macrophages was done using the above media plus 50 ng/ml IL6 [3] in normal adherence plates. IL6 was added to infected samples at 10 dpi. All samples used were derived from HCMV negative donors which was confirmed by nested PCR for viral DNA using primers IE 2A with IE 4B for the first PCR, and IE 3A with IE 3B for the nested reaction (Table 1). The FIX-WT, FIX-ΔLUNA, and FIX-Rev viruses were propagated in HF cells, and viral titers were determined using standard plaque assays [37]. For infection, all cells were incubated with FIX-WT, FIX-ΔLUNA, or FIX-Rev in DMEM at a multiplicity of infection (MOI) of 1 or 3 (where indicated) for 1 hr at 37°C. Following the infection, cells were washed twice with 1× PBS to remove any unabsorbed virus. Virus production was monitored by plaque assay.

### Flow Cytometry

Purity of the CD14^+^ cell population was tested using flow cytometry. A total of 1×10^5^ cells were resuspended in 100 µl 1× PBS and incubated for 15 min at RT in the dark with 10 µl mouse anti-human CD14 PE antibody (BD Bioscience) or mouse IgG2b PE antibody as an isotype control (BD Bioscience). Cells were then washed twice with 2 ml of 1× PBS, then resuspended in PBS plus 0.1% Na Azide, and spun for 5 min at 1500 rpm. Cells were finally resuspended in 500 µl of 1% formaldehyde and stored at +4°C until use. The FACSort system with CELLQUEST software (Becton Dickinson) was used to analyze the cells. Forward and side-scatter plots were used to exclude dead cells/debris from the histogram analysis plots.

### BAC Recombineering

The FIX-BAC-ΔLUNA and FIX-BAC-Rev viruses were constructed via BAC mutagenesis and homologous recombination as previously described [24]. Briefly, to target the site within the FIX-BAC genome we intended to mutate, we amplified PCR products using a pGalK-Kan cassette and primers flanking our target site, the beginning of UL81-82ast, using primers from Table 1. The addition and replacement of the cassette and addition of the mutations/reversions were confirmed by a restriction digest, southern blotting, and sequence analysis. The recombinant BACs were then electroporated into HF cells to propagate the virus.

### Southern Blot analysis

The digested BAC DNA was separated out on a 0.5% agarose gel, which was then treated with 0.25M HCl for 10 min and rinsed with diH_2_O. The DNA was transferred onto a nylon membrane overnight via capillary blotting in 0.4M NaOH. The GalK probe was made by PCR using GalK forward and reverse primers (Table 1) and labeled using Rediprime II DNA labeling system (GE Healthcare) following manufacturer's recommendations. The bands were then detected using a phospo-imager screen and an Amersham Storm Scanner.

### Immunofluorescence Microscopy

HF cells and CD14^+^ cells were infected with FIX-WT, FIX-ΔLUNA, and FIX-Rev. Infected cells were fixed at 1 dpi on glass cover slips with 4× paraformaldehyde (PFA) for 1 hr at +4°C. The infected CD14^+^ cells were prepared by cytocentrifugation using a Shandon Elliott Cytospin at 400 rpm for 5 min onto ProbeOn Plus slides (Fisher Scientific, Pittsburgh, Pa.), and re-fixed with 1× PFA for 1 hr at +4°C. All cells were blocked with 10% normal goat serum (NGS) for 1 hr at RT to prevent non-specific antibody binding, then incubated with the primary antibodies anti-LUNA pre-absorbed rabbit serum, (1∶500 dilution) and mouse anti-IE (Millipore, 1∶250) in 2% NGS overnight. Cells were washed three times with 1× PBS and next incubated with secondary antibodies Alexa Fluor 594 (red) goat anti-rabbit IgG (dilution 1∶1000), and Alexa Fluor 488 (green) goat anti-mouse IgG (dilution 1∶1000) (Molecular Probes, Inc.) for 1 hr at RT, washed and then treated with DAPI for 30 min RT. Slides were fixed with cytoseal 60 (Thermo Scientific).

### DNA, RNA and Protein analysis

Using Illustra™ triplePrep kit (VWR) we were able to isolate DNA, RNA, and Protein from the same sample following manufacturer recommendations. During RNA isolation, samples were subjected to on-column DNase treatment during RNA isolation. cDNA was synthesized using SuperScript™ III Reverse Transcriptase (Invitrogen) following manufacturer's recommendations. To determine viral genome copy number and changes in viral gene expression qPCR and RT-PCR was performed respectively. qPCR was performed on a 7500 Fast Real-Time PCR System (Applied Biosystems) using FastSYBR® Green. Viral genome copies were normalized to cellular genes PPIA, and GapDH. All primers used are listed in Table 1.

### Quantitative Real-Time PCR

In order to analyze levels of viral gene expression, total RNA was extracted from 5×10^5^ cells using Trizol (Invitrogen) per manufactures' instructions and the resultant pellet was resuspended in 50 µL of nuclease free water. The isolated RNA was DNAse-treated using DNA-free (Ambion). Reverse transcription (RT) of mRNA was performed on 1 µg of total RNA with murine leukemia virus (MuLV) reverse transcriptase by priming with Oligo d(T)_16_ using the GeneAmp RNA PCR Kit (Applied Biosystems) according to the manufactures' instructions. Following RT, 1 µl of cDNA from each sample was amplified using primers (Table 1) for UL138, US28, vIL-10, and β-actin (10 pmol) in a SybrGreen PCR master mix (Applied Biosystems) and analyzed on a 7900 quantitative PCR machine (ABI). Standards were derived from serial dilutions of FIX-BAC DNA containing β-actin (Andrew Womack and Tom Shenk, unpublished), using the following formula: # of molecules = moles of DNA×6.022×10^23^. In our system, 2.49 µg/µL of FIX BAC actin DNA is approximately 10^10^ molecules/µL.

### Cell Dilution Assay

The number of viral genomes present prior to IL-6 induced differentiation/reactivation were determined using a standard cell dilution assay. Intracellular viral DNA and DNA from cell supernatants were assayed by qPCR using primers for the HCMV UL123 ORF and β-actin. Cells were infected at an MOI of 1 for 1 hr, then counted and added to a 96 well plate. 5 fold dilutions were made and cells were lysed in the dish via boiling. qPCR was performed using 2X SYBR-GREEN master mix (Applied Biosciences) and primers specific for UL123 and β-actin, samples were tested in triplicate. UL123 quantities were standardized against β-actin to normalize for cell number. Viral quantities were calculated against a standard curve of viral DNA.

### Infectious Units (IU)/Reactivation Assay

The yield of virus produced by cells was assayed by adding lysed cells and supernatants onto fibroblasts and quantifying the number of IE-1 expressing cells by IFA 48 hr later, as previously described [3], [4], [38], [39]. Briefly, supernatant and cell lysates from FIX-WT, FIX-ΔLUNA and FIX-Rev infected CD14^+^ cells were collected at different times post infection. Cell lysate and supernatant for each condition were mixed and serially diluted prior to infection of HF cells in a 96 well plate for 1 hr. Cells were then washed twice with 1× PBS and left to grow under normal conditions for 48 hr. Following the 48 hrs, cells were fixed with ice cold methanol for 15 min at −20°C. The cells were then stained with anti-IE1/2 antibodies as described for IFA above. Each IE positive cells was counted as one infectious unit (IU) and IU/ml was calculated by dividing the number of IE positive cells by the dilution factor and multiplying by 1000.

## Supporting Information

Figure S1 **Purity of Isolated CD14^+^ monocytes.** Isolated CD14+ cells were analyzed for extracellular CD14 marker by flow cytometry. Histograms are shown for CD14+ cells stained with isotype control (grey line) or CD14 antibody (black line).
(TIF)

Figure S2 **Availability of genomes prior to reactivation as determined by cell dilution PCR.** Intracellular viral DNA and DNA from cell supernatants was extracted at 10 dpi and were assayed by qPCR using primers for the HCMV UL123 ORF and b-actin. Cells were infected at an MOI of 1 for 1 hr. Cells were counted and added to a 96 well plate. 5 fold dilutions were made and cells were lysed in the dish by boiling. 2X SYBR-GREEN master mix (Applied Biosciences) and primers were added to the mixture and triplicates were analyzed for UL123 and B-actin. Amounts of UL123 were standardized against b-actin to normalize for cell number. Viral quantities were calculated against a standard curve of viral DNA.
(TIF)
