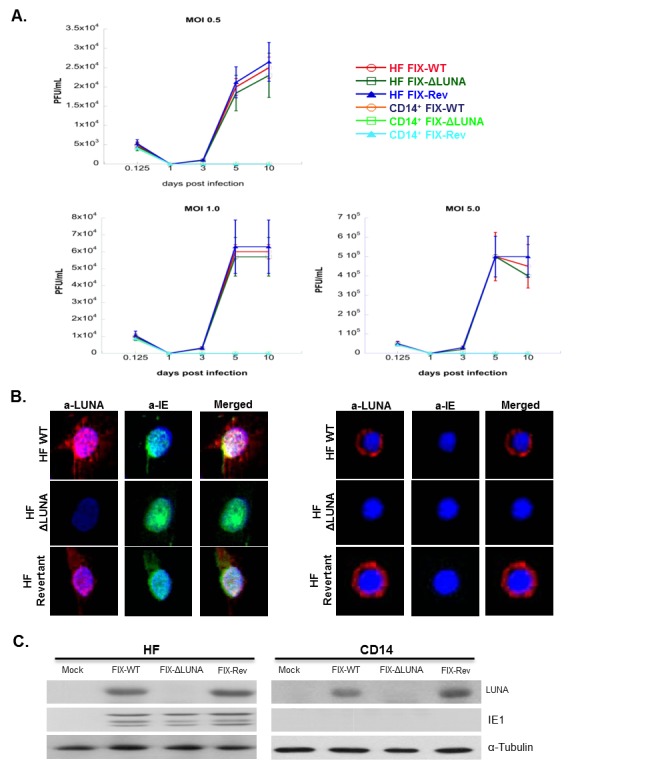# Correction: HCMV Protein LUNA Is Required for Viral Reactivation from Latently Infected Primary CD14^+^ Cells

**DOI:** 10.1371/annotation/ecd7e767-0a1b-46ee-afa8-8f29ae9c815d

**Published:** 2013-10-10

**Authors:** Lisa R. Keyes, Danna Hargett, Melisa Soland, Mariana G. Bego, Cyprian C. Rossetto, Graca Almeida-Porada, Stephen St. Jeor

In Figure 2, panel C, one of the western blots was switched. The actin control and the a-IE blot were inverted. Please see the corrected Figure 2 here: 

**Figure:**