# Supplementary figures and images for: HCMV Protein LUNA Is Required for Viral Reactivation from Latently Infected Primary CD14+ Cells
Source: PLoS One. 2012 Dec 26;7(12):e52827. doi: 10.1371/journal.pone.0052827 (PMC3530514; doi:10.1371/journal.pone.0052827)

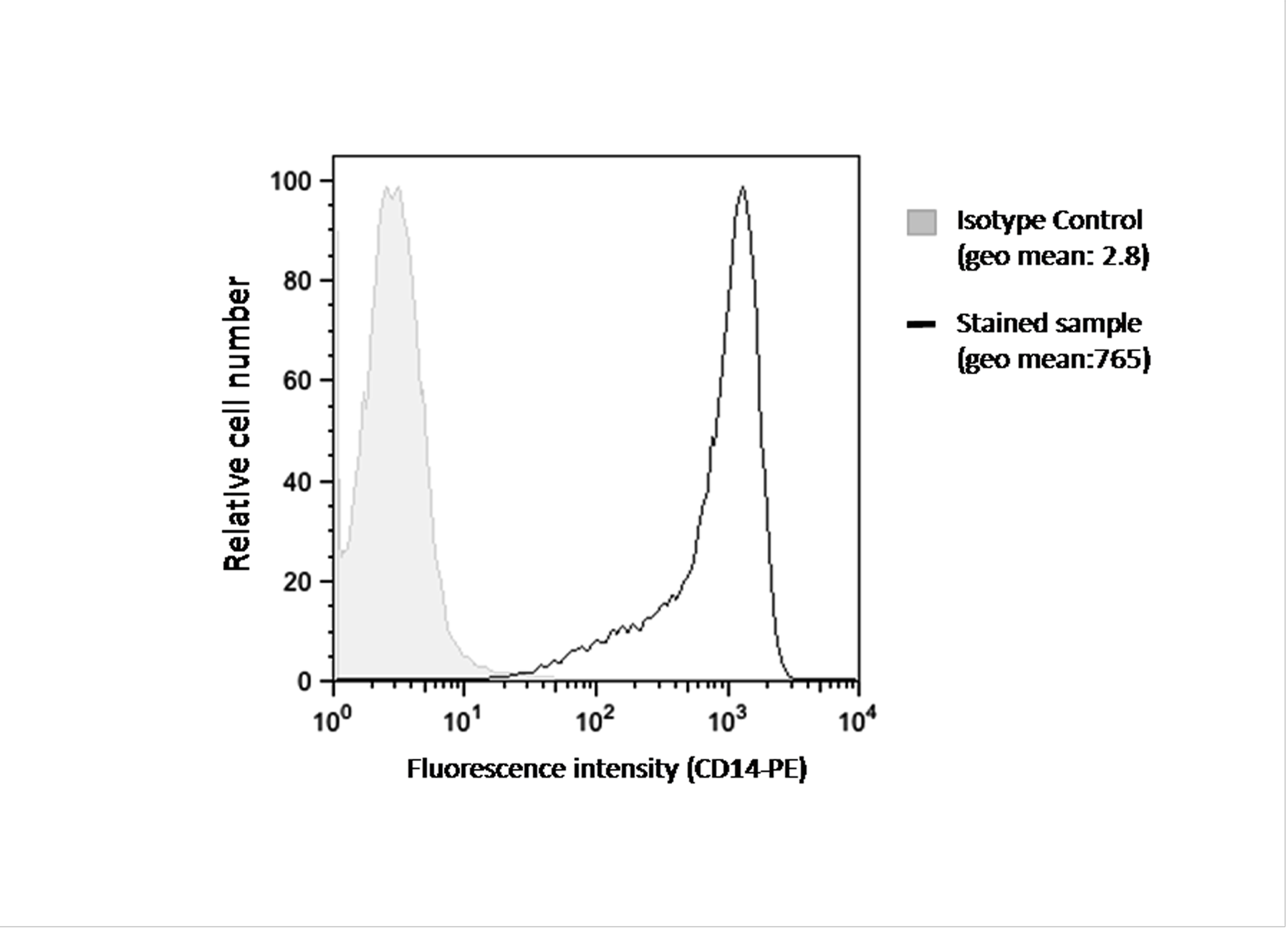

Supplement: Figure S1 — Purity of Isolated CD14+ monocytes. Isolated CD14+ cells were analyzed for extracellular CD14 marker by flow cytometry. Histograms are shown for CD14+ cells stained with isotype control (grey line) or CD14 antibody (black line). (TIF) [file pone.0052827.s001.tif]

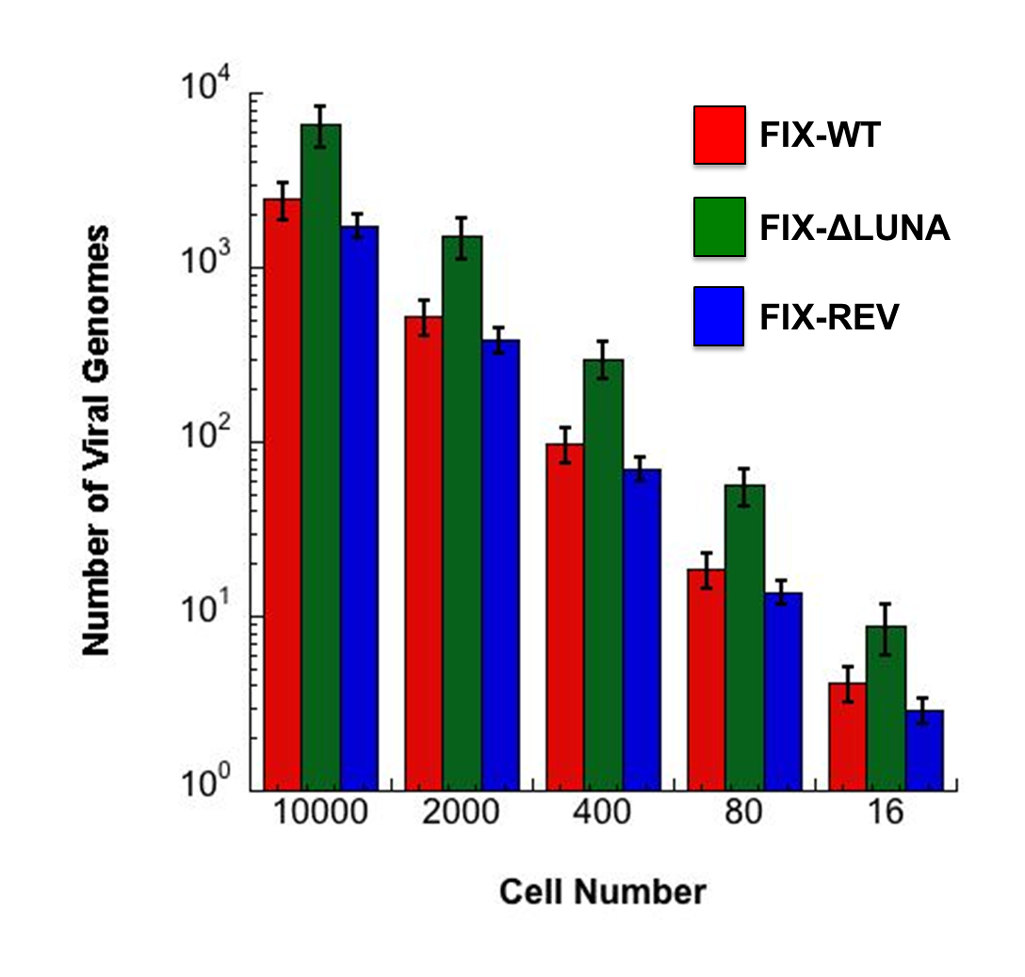

Supplement: Figure S2 — Availability of genomes prior to reactivation as determined by cell dilution PCR. Intracellular viral DNA and DNA from cell supernatants was extracted at 10 dpi and were assayed by qPCR using primers for the HCMV UL123 ORF and b-actin. Cells were infected at an MOI of 1 for 1 hr. Cells were counted and added to a 96 well plate. 5 fold dilutions were made and cells were lysed in the dish by boiling. 2X SYBR-GREEN master mix (Applied Biosciences) and primers were added to the mixture and triplicates were analyzed for UL123 and B-actin. Amounts of UL123 were standardized against b-actin to normalize for cell number. Viral quantities were calculated against a standard curve of viral DNA. (TIF) [file pone.0052827.s002.tif]
